# Converting cyclosporine A from intravenous to oral administration in hematopoietic stem cell transplant recipients and the role of azole antifungals

**DOI:** 10.1007/s00228-018-2434-4

**Published:** 2018-03-02

**Authors:** Ferdows Atiq, Edon Hameli, Annoek E. C. Broers, Jeanette K. Doorduijn, Teun Van Gelder, Louise M. Andrews, Birgit C. P. Koch, Jorie Versmissen, Brenda C. M. de Winter

**Affiliations:** 1 Department of Hospital Pharmacy, Erasmus Medical Center, PO Box 2040 3000, CA Rotterdam, The Netherlands; 2 Department of Hematology, Erasmus Medical Center, Rotterdam, The Netherlands; 3 Department of Internal Medicine, Erasmus Medical Center, Rotterdam, The Netherlands

**Keywords:** Cyclosporine A, Immunosuppression, Hematopoietic stem cell transplantation, Dose conversion, Ferdows Atiq and Edon Hameli contributed equally to this work.

## Abstract

**Purpose:**

Cyclosporine A (CsA) is the most widely used immunosuppressive agent after a hematopoietic stem cell transplantation (HSCT). Although recommendations for CsA dose conversion from intravenous to oral administration differ from 1:1 to 1:3, most studies did not consider the role of azole antifungals as an important confounder. Therefore, we assess the optimal conversion rate of CsA from intravenous to oral administration in HSCT recipients, taking into account the concomitant use of azole antifungals.

**Methods:**

We retrospectively included patients from a large database of 483 patients who underwent a HSCT and received intravenous CsA as part of the conditioning regimen and peritransplant immunosuppression. All patients were converted from intravenous to oral administration in a 1:1 conversion rate. We collected for each patient three CsA trough concentrations during intravenous and oral administration, directly before and after conversion to oral administration.

**Results:**

We included 71 patients; 50 patients co-treated with fluconazole, 10 with voriconazole, and 11 without azole co-medication. In patients with voriconazole, the dose-corrected CsA concentration (CsA concentration divided by CsA dosage) was not different between intravenous and oral administration (2.6% difference, *p* = 0.754), suggesting a CsA oral bioavailability of nearly 100%. In patients with fluconazole and without azole co-medication, the dose-corrected CsA concentration was respectively 21.5% (*p* < 0.001) and 25.2% (*p* = 0.069) lower during oral administration.

**Conclusions:**

In patients with voriconazole, CsA should be converted 1:1 from intravenous to oral administration. In patients with fluconazole and without azole co-medication, a 1:1.3 substitution is advised to prevent subtherapeutic CsA concentrations.

## Introduction

Cyclosporine A (CsA) is the most widely used immunosuppressive drug in the prevention and treatment of graft-versus-host disease (GvHD) after hematopoietic stem cell transplantation (HSCT) [1–4]. CsA is a narrow therapeutic index drug, and blood levels are routinely monitored to maintain a therapeutic drug concentration [1, 5]. Suboptimal CsA concentrations in the early period after HSCT are associated with a higher incidence of acute GvHD [6]. Development of moderate to severe GvHD is associated with higher early mortality after HSCT [6]. The most relevant side effects of CsA are nephro- and hepatotoxicity, which are correlated with high CsA concentrations [2].

CsA is absorbed in the ileum and jejunum [1]. CsA has a large inter- and intra-individual variability in bioavailability, which depends on food intake, time since transplantation, liver function, bile flow, genetics, and gastrointestinal state such as motility and length of the small intestine and diarrhea (which can decrease the absorption of CsA) [1, 7].

In the early period after, HSCT patients can have a damaged oral and gastrointestinal mucosa, especially after myeloablative conditioning. Other patients experience difficulty in swallowing tablets due to nausea after the conditioning regimen. In these patients, CsA is administered intravenously to ensure that therapeutic concentrations are reached [3]. As soon as patients tolerate oral intake, CsA is switched from intravenous to oral administration [3]. The optimal conversion rate from intravenous to oral administration is not unambiguous due to differences in absorption and first pass effect during oral administration [8]. Recommendations for CsA conversion in HSCT patients from intravenous to oral administration differ from a conversion rate of 1:1 to 1:3 [9–13]. However, recent studies recommend a conversion rate of 1:2 from intravenous to oral administration [11–13].

CsA is a CYP3A4 and P-glycoprotein (P-gp) substrate and may therefore be influenced by CYP3A4 and P-gp inhibitors and inducers [1, 14]. Azole antifungals, which are commonly used in the post-transplant period, increase CsA concentrations by these mechanisms [15]. Azole antifungals can influence the bioavailability of CsA and therefore are likely to play a role in the dose conversion of CsA. A study in four patients found that concomitant use of voriconazole increased CsA bioavailability, suggesting that a lower conversion rate from intravenous to oral administration may be better in patients taking oral voriconazole [13].

The aim of this study was to assess the optimal conversion rate of CsA from intravenous to oral administration in HSCT recipients, taking into account the concomitant use of different azole antifungals.

## Materials and methods

We retrospectively included patients from a large database of 483 patients who underwent a HSCT from 2008 until 2014 in the Erasmus Medical Center. Patients receiving intravenous CsA as part of the conditioning regimen and peritransplant immunosuppression were included. Furthermore, at least one conversion of CsA from the initial intravenous therapy to oral administration had to be performed. The applied dose conversion rate in all patients was 1:1. During both intravenous and oral administration, CsA (Neoral®, Novartis) was administered twice daily. CsA dosage was initiated based on patients’ weight. After measuring CsA concentrations, the CsA dose was modified to remain within the therapeutic range.

To monitor the CsA concentration before and after the conversion and to take the CsA intrapatient variability into account, we aimed to collect for each patient three CsA trough concentrations during intravenous administration and three trough concentrations during oral administration. The collected concentrations were taken directly before and after the conversion to oral administration. The CsA level was routinely measured 3 days/week, on Monday, Wednesday, and Friday during hospital stay. After discharge from hospital, CsA measurement was planned weekly. The CsA target level was 250–350 ng/mL. The CsA assay was performed using a validated UPLC-MS/MS method, routinely used in our clinic. This method was developed in-house and validated according to the FDA guidelines.

In our center, patients with an expected neutropenia (granulocytes < 0.5 × 10^9^/L) lasting longer than 10 days received antimicrobial and antifungal drugs until neutrophil recovery. Antifungal drugs were preferred to be administered orally. If patients could not tolerate oral intake, antifungal drugs were administered intravenously. The standard antifungal treatment was fluconazole 400 mg/day. Patients with a history of invasive aspergillosis receive voriconazole as secondary prophylaxis, until immunosuppressive treatment is discontinued.

In order to assess the influence of co-treatment with azole antifungal drugs, patients were only included if the azole antifungal was used during all CsA concentration measurements. If a particular antifungal drug was used in less than five patients, these patients were excluded.

All patients had signed informed consent at the time of HSCT, which allowed for additional analyses on anonymized data (such as the present retrospective analysis).

### Clinical parameters

We assessed the influence of diarrhea and oral mucositis on CsA concentrations. A patient was defined as having diarrhea or mucositis when either of them was documented by physicians during at least one of the CsA concentration measurements. We also assessed the influence of CsA dose conversion on renal function. The glomerular filtration rate (eGFR) was calculated using the Modification of Diet in Renal Diseases (MDRD) equation: eGFR (ml/min/1.73 m2) = 186 × (serum creatinine (μmol/l) / 88.4) − 1.154 × age (in years)–0.203 × 0.742 (for women) and multiplied by 1.21 for black patients.

### Analysis

We categorized patients based on the use of concomitant azole antifungal drugs. We calculated the dose-corrected CsA concentration for each measurement by dividing the CsA concentration by the CsA dosage (daily dose) used at that time point. The average dose-corrected CsA concentration was calculated from the three concentrations measured for each patient during intravenous and oral administration separately. The average CsA concentrations per patient during intravenous and oral administration were normally distributed. Therefore, we compared the mean calculated dose-corrected CsA concentrations between the intravenous and oral period for each antifungal group by using a paired *t* test. We calculated the bioavailability by dividing the mean dose-corrected CsA concentration during oral administration by the mean dose-corrected CsA concentration during intravenous administration. We compared groups by using an unpaired *t* test. We used an unpaired *t* test for continuous baseline characteristics and chi-square for categorical variables. We compared categorical variables between groups by using a chi-square test. A *p* value below 0.05 was considered to be significant.

## Results

**Fig. 1:**
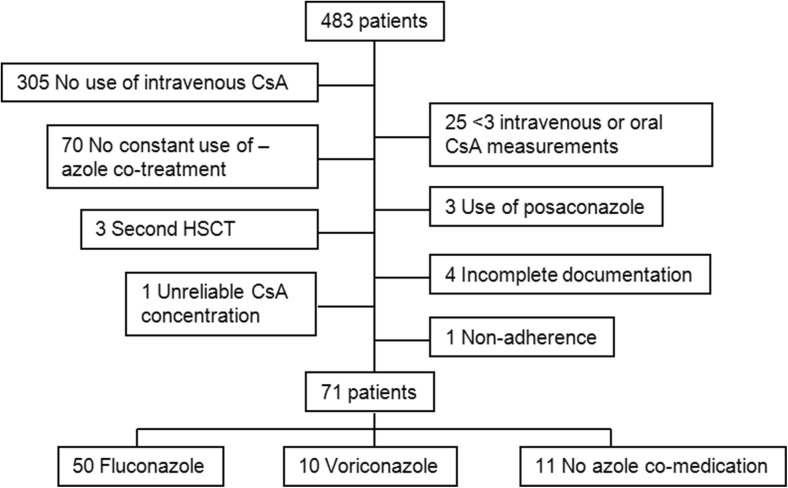
Consort diagram. HSCT hematopoietic stem cell transplantation, IV1, IV2, IV3 first, second, last intravenous CsA administration before dose conversion, PO1, PO2, PO3 first, second, third oral administration after dose conversion

We included 71 out of 483 transplanted patients. Figure 1 shows the reasons for exclusion. Most patients were excluded because they were not treated with intravenous CsA. A second reason for excluding patients was the lack of treatment with azole antifungal drugs for the full period during which the three intravenous and three oral CsA concentrations were obtained. Posaconazole-treated patients were excluded from the analysis due to insufficient numbers. One patient was excluded because of unreliable CsA concentrations with a mean dose-corrected CsA concentration that was exceptionally high (7.31 vs group mean of 2.05 ± 0.77). None of the included patients used other co-medication which had an interaction with CsA.

Patient characteristics are presented for the total dataset and for the three antifungal groups separately (Table 1). The mean age was significantly higher in patients with fluconazole compared to patients with voriconazole (*p* = 0.019) and patients without azole co-medication (*p* = 0.035). No other differences in baseline characteristics existed between the different groups. Overall, the number of days between the first and the last CsA measurement was significantly longer during oral administration than during intravenous administration (*p* = 0.034). This is explained by discharge from the hospital and less frequent measurements subsequently. Most patients were diagnosed with acute myeloid leukemia (AML), followed by myeloproliferative syndrome (MPS) (Table 2). All patients received peripheral blood stem cells except for one patient in the voriconazole group who received bone marrow-derived stem cells. Seventy percent of patients received cord blood-derived stem cells preceded by reduced intensity conditioning (RIC), which consisted of a single administration of cyclophosphamide followed by 4 days of fludarabine and 2 days of total body irradiation (2 Gy TBI). Twenty-one percent of patients had a matched unrelated donor (MUD) and 9% had a sibling donor. Ten patients received myeloablative conditioning (MAC), consisting of high-dose cyclophosphamide and 2 × 6 Gy TBI or cyclophosphamide combined with busulfan. Six patients received RIC, consisting of fludarabine and 2 Gy TBI. Five patients received a conditioning regimen with high-dose post-transplant cyclophosphamide. In almost all patients (98%), fluconazole was administered once daily, whereas in all patients, voriconazole was administered twice daily. The mean daily fluconazole and voriconazole doses were respectively 382 mg (± 7.92) and 457 mg (± 39.8).

**Table 1:** Patient characteristics

	Patients	Female	Age (year)	Weight (kg)	Length (m)	Number of days between		
						IV1-IV3	IV3-PO1	PO1-PO3
Fluconazole	50	18 (39%)	52 (22–70)	77 (53–107)	1.76 (1.51–1.96)	4.80* (3–7)	2.26 (1–4)	4.98* (3–7)
Voriconazole	10	4 (40%)	41 (19–65)	69 (58–94)	1.75 (1.59–1.95)	5.10** (4–7)	3.10 (1–8)	5.20** (4–9)
No azole	11	6 (55%)	43 (21–69)	75 (58–108)	1.75 (1.61–1.88)	4.73*** (4–5)	2.36 (2–4)	6.55*** (5–14)
Total	71	29 (41%)	49 (19–70)	76 (53–108)	1.76 (1.51–1.96)	4.83# (3–7)	2.39 (1–8)	5.25# (3–14)

Numbers are given as mean (range) or number (%)

*IV1-IV3* first and last intravenous concentration, *IV3-PO1* last intravenous and first oral administration, *PO1-PO3* first and last oral administration

*p = 0.253; **p = 0.859; ***p = 0.056; #*p* = 0.034

**Table 2:** Patients diagnosis and treatment

	Diagnosis										Donor			Conditioning	
	ALL	AML	CLL	CML	MDS	MPS	NHL	PCL	PLL	SAA	CB	MUD	Sib	RIC	MAC
Fluconazole	2	21	5	4	4	7	5	2	–	–	74%	20%	6%	92%	8%
Voriconazole	–	6	1	–	1	–	–	–	1	1	70%	20%	10%	70%	30%
No azole	–	3	–	1	2	3	2	–	–	–	55%	27%	18%	73%	27%
Total	2	30	6	5	7	10	7	2	1	1	70%	21%	8%	86%	14%

*ALL* acute lymphatic leukemia, *AML* acute myeloid leukemia, *CLL* chronic lymphatic leukemia, *CML* chronic myeloid leukemia, *MDS* myeloid dysplastic syndrome, *MPS* myeloproliferative syndrome, *NHL* non-Hodgkin lymphoma, *PCL* plasmacell leukemia, *PLL* prolymphocytic leukemia, *SAA* severe aplastic anemia, *CB* cord blood, *MUD* matched unrelated donor, *Sib* sibling, *RIC* reduced intensity conditioning, *MAC* myeloablative conditioning

**Fig. 2:**
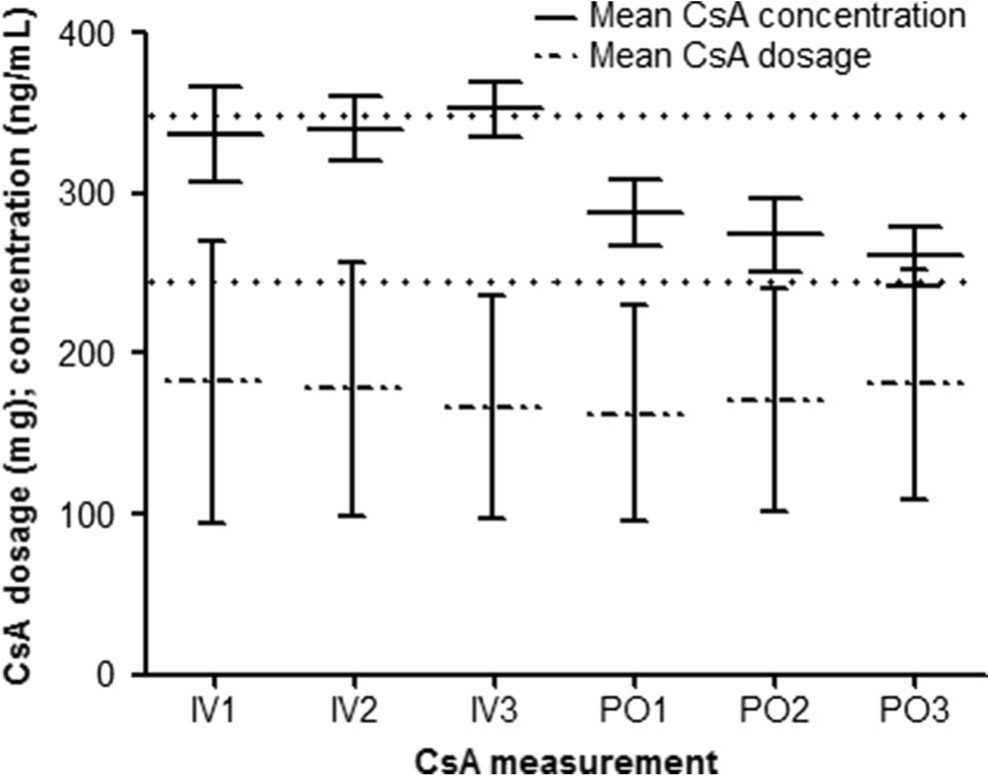
Mean CsA concentration and dosage. Data are presented as mean with 95% confidence interval. The dotted lines at 250 and 350 reflect the therapeutic range of CsA. IV1, IV2, IV3 first, second, last intravenous CsA administration before dose conversion, PO1, PO2, PO3 first, second, third oral administration after dose conversion

In the total population, compared to intravenous CsA administration, the mean CsA concentration was 20% lower during oral administration (*p* < 0.001) (Fig. 2). In patients with fluconazole and in patients without azole co-medication, the mean CsA concentration was lower during oral administration, 19% (*p* < 0.001) and 31% (*p* = 0.014), respectively. However, in patients with voriconazole co-treatment, there was no difference in mean CsA concentrations during intravenous and oral administration (339.9 ± 24.5 vs 302.5 ± 29.0 ng/mL; *p* = 0.175). For the total of patients, there was no difference in the mean CsA dosage during intravenous and oral administration (175.4 mg ± 8.90 vs 171.5 mg ± 7.57, *p* = 0.476). In patients with fluconazole, voriconazole, and without azole co-medication, the average CsA daily doses during intravenous compared to oral administration were respectively 170.6 mg ± 8.63 vs 174.5 mg ± 8.95 (*p* = 0.428), 134.1 mg ± 18.0 vs 117.5 mg ± 12.7 (*p* = 0.125), and 234.8 mg ± 33.7 vs 206.8 mg ± 16.7 (*p* = 0.300).

**Fig. 3:**
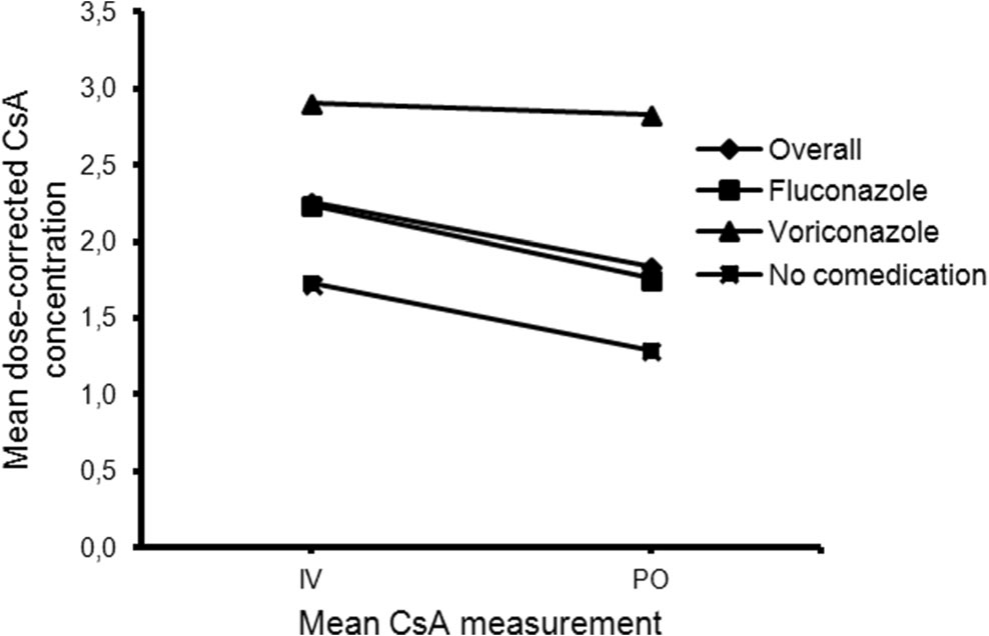
Mean dose-corrected CsA concentration. Each symbol reflects the mean dose-corrected CsA concentration at the illustrated time point

In all patients, the mean dose-corrected CsA concentration was 18.5% (*p* < 0.001) lower during oral administration (Fig. 3). In patients with voriconazole co-treatment, there was no difference in dose-corrected CsA concentrations during intravenous and oral administration (2.6% lower during oral administration, *p* = 0.754), whereas in patients with fluconazole and without azole co-medication, the dose-corrected CsA concentration was respectively 21.5% (*p* < 0.001) and 25.2% (*p* = 0.069) lower during oral administration. Patients with fluconazole showed a similar trend as patients without azole co-medication (Fig. 3); therefore, these patients were grouped together.

**Fig. 4:**
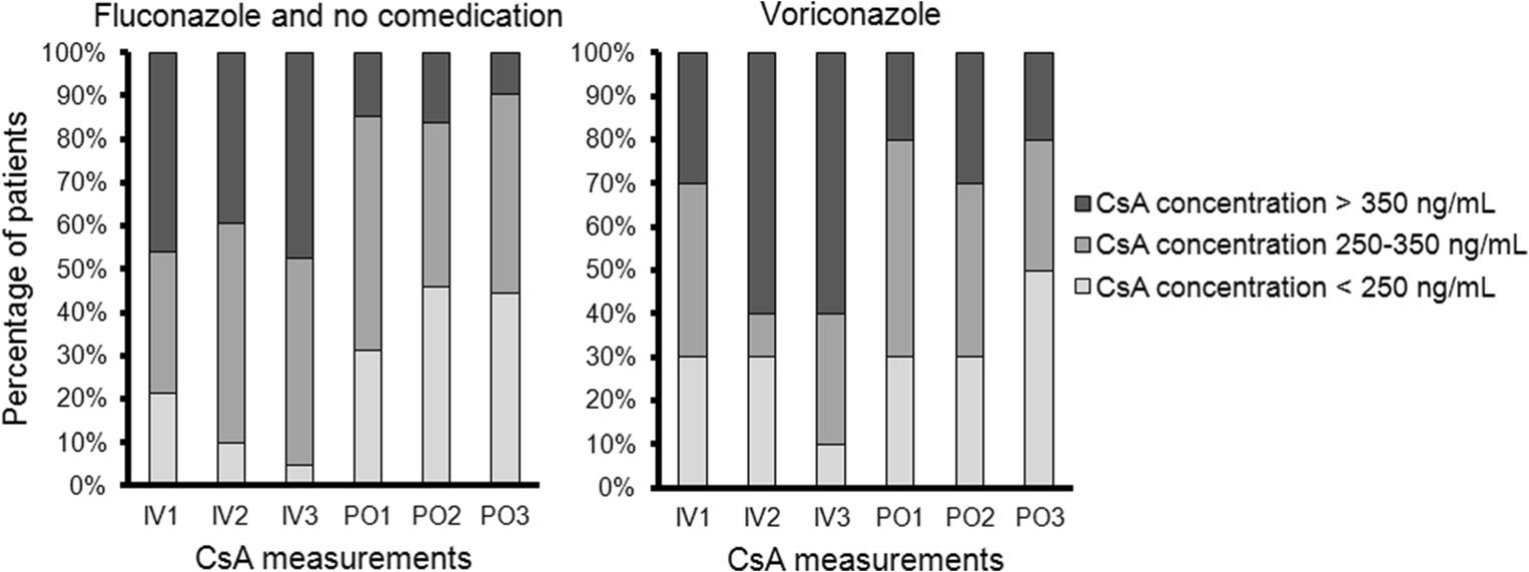
Distribution of CsA concentrations. Percentage of patients with subtherapeutic, therapeutic, and supratherapeutic CsA concentrations. IV1, IV2, IV3 first, second, last intravenous CsA administration before dose conversion, PO1, PO2, PO3 first, second, third oral administration after dose conversion

CsA concentrations were often supratherapeutic during intravenous administration, while they were more often subtherapeutic during oral administration (Fig. 4). The proportion of patients with therapeutic CsA concentrations remained nearly equal, while mean CsA concentrations decreased, indicating a shift from high to low CsA concentrations.

### Diarrhea and oral mucositis

In this series of patients, diarrhea was present in 14% of the patients. The dose-corrected CsA concentration was not related with the presence of diarrhea.

Oral mucositis was present in 22% of patients on fluconazole, in 40% of patients on voriconazole, and in 45% of patients without azole co-treatment. In patients on fluconazole, the bioavailability was lower in patients with mucositis compared to patients without mucositis (0.66 ± 0.06 vs 0.90 ± 0.05, *p* = 0.035). In patients on voriconazole and without azole co-medication, there was no significant difference in the CsA concentration between patients with and without oral mucositis.

### Renal function

**Fig. 5:**
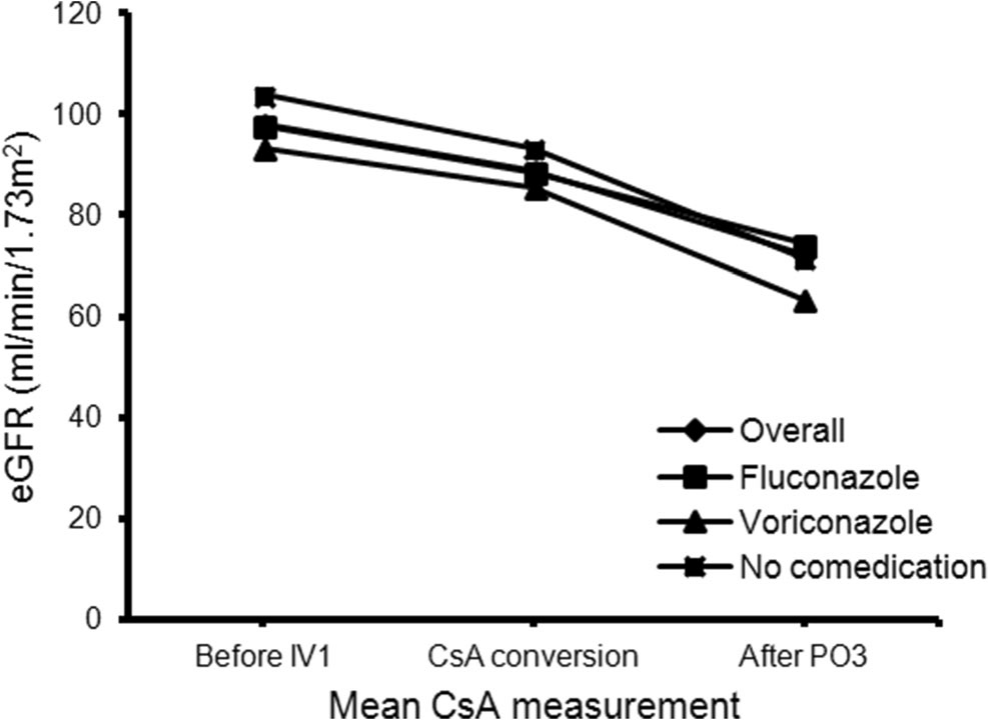
Change in eGFR. Each symbol reflects the eGFR at the illustrated time point. eGFR glomerular filtration rate, before IV1 before the first included intravenous CsA administration, after PO3 after the last included oral CsA concentration

The eGFR significantly decreased during intravenous and oral administration (respectively with Δ9.2 ± 27.9 mL/min, *p* = 0.007 and Δ16.6 ± 29.9 mL/min, *p* < 0.001). There was no difference in number of days between first and last creatinine measurement (*p* = 0.168) during intravenous and oral administration (respectively 7.4 ± 1.7 and 8.1 ± 3.9 days). The decrease of eGFR was similar during intravenous and oral administration (*p* = 0.088) (Fig. 5).

## Discussion

In this study, we show that the bioavailability of CsA in HSCT patients is remarkably high (75–80%), especially in patients co-treated with voriconazole (almost 100%). This is quite surprising compared to current available literature [11, 13–15]. In our center, hematologists have used a 1:1 ratio for switching CsA from intravenous to oral administration, and the data shown in this paper support that strategy, especially for the group of voriconazole co-treated patients [10]. In patients co-treated with fluconazole and in patients without azole co-treatment, a conversion rate of 1: 1.3 should allow for maintaining stable CsA exposure. The CsA dose conversion did not influence the decrease of renal function caused by CsA administration.

Our results are partially in accordance with earlier studies in which the recommendation for dose conversion rate in HSCT patients differed from 1:1 to 1:3. Nevertheless, there are major differences in study design and study subjects. Choi et al. showed in 33 pediatric patients that CsA concentrations remained in the therapeutic range with a conversion rate of 1:3 from intravenous to oral administration [9]. They found a bioavailability of 0.43 during oral CsA administration. However, this study was in children and may not be applicable to adults. Moreover, information on co-medication was limited. Parquet et al. showed in a randomized controlled trial in 14 patients comparing 1:1 with 1:2 conversion that the converting rate of 1:2 tended to give more therapeutic CsA concentrations in patients with concomitant use of fluconazole [11]. Inoue et al. prospectively studied 11 patients that were converted in a 1:2 ratio and found that the area under the curve (AUC) remained nearly the same during intravenous and oral administration [12]. Patients were co-treated with micafungin, a non-azole antifungal agent, known to be a mild CYP3A4 inhibitor. The mean bioavailability of CsA during oral administration was 0.58 ± 0.15. Kimura et al. investigated in 12 patients the conversion of CsA from continuous intravenous administration to oral administration in a 1:2 conversion rate [13]. AUC during oral administration was significantly higher than the AUC during intravenous infusion (median 7508 vs 6705 ng/ml h, *p* = 0.050). The bioavailability of CsA was 0.685 (range 0.45–1.04), but concomitant administration of voriconazole (*n* = 4) significantly increased the bioavailability of CsA (median 0.87 vs 0.54, *p* = 0.017). McGuire et al. found in 52 patients with a dose conversion rate of 1:3 that the incidence of renal dysfunction during intravenous and oral administration was respectively 29 and 63% (*p* = 0.0018) [10]. Therefore, they suggested a dose conversion of 1:1 as more appropriate to avoid renal dysfunction. However, they did not measure CsA concentrations and it is not clear whether the higher incidence of renal dysfunction was due to the CsA conversion from intravenous to oral or because patients with oral administration used CsA for a longer period of time. Moreover, it is unclear whether the patients used azole antifungals.

To summarize, the published studies on CsA dose conversion included small numbers of patients and often did not take the role of azole antifungals into account. These studies showed that the bioavailability of CsA (Neoral®) in adults ranged between 0.58 and 0.685, and therefore, a dose conversion rate between 1:1 and 1:2 would be appropriate, as also found in our study.

For voriconazole, which is a stronger CYP3A4 inhibitor, the dose-corrected CsA concentration is nearly the same during intravenous and oral administration, suggesting that voriconazole co-treatment increases the oral bioavailability of CsA to almost 100%. This is in accordance with the study of Kimura et al. [13]. This is probably caused by inhibition of CYP3A4 enzymes in the gut wall by voriconazole. This finding was not observed in patients with fluconazole which is a less potent CYP3A4 inhibitor [16].

The presence of mucositis resulted in a lower bioavailability in patients co-treated with fluconazole, which may be caused directly by a lower CsA absorption or indirectly by a lower fluconazole absorption as shown earlier for posaconazole [15]. There was no influence of mucositis on CsA concentration in patients with voriconazole or without azole co-medication, most likely due to the small number of patients.

Strengths of our study are the large number of patients compared to previous reports, the focus on the role of azole antifungals on the CsA dose conversion, and the detailed recommendations we provide for dose conversion. A potential limitation is the retrospective observational study design. However, the medication data and laboratory measurements were well documented in the patient files. Another potential limitation is that we could not assess dose conversion during use of other azole antifungals than voriconazole and fluconazole. The dose conversion rate for other azole antifungals could be extrapolated based on the degree of CYP3A4 inhibition of other azole antifungals. Ketoconazole, itraconazole, and miconazole are known to be strong CYP3A4 inhibitors and therefore CsA conversion with co-treatment of these azoles may be comparable with CsA conversion during voriconazole co-treatment [17]. We did not calculate AUCs which would have made comparison with earlier studies more reliable. However, for clinical use and recommendations trough levels are the most important parameters of CsA pharmacokinetics. Furthermore, although our total sample size is large compared to previous studies on the dose conversion of CsA, the subgroups (i.e., voriconazole, no azole co-medication) are rather small. However, we do not think that our conclusion is influenced by the sample size, because in patients co-treated with voriconazole, the difference in dose-corrected CsA concentration between intravenous and oral administration was small (Fig. 3).

In conclusion, we found that CsA dose conversion from intravenous to oral administration depends on co-treatment with azole antifungal drugs. In patients co-treated with voriconazole, the bioavailability of orally administered CsA is almost 100%, and thus 1:1 conversion is best. During co-treatment with fluconazole or without azole co-medication, the CsA concentrations will drop with 20–25% after conversion from intravenous to oral administration. Therefore, we recommend a dose conversion rate of 1:1.3 in these patients.
